# From Molecular Visualization to Spatial Landscapes: Engineering the Next Generation of In Situ Hybridization

**DOI:** 10.3390/genes17060616

**Published:** 2026-05-29

**Authors:** Zejia Li, Miaomiao Luo, Minshuai Zhu, Yun Bai

**Affiliations:** 1 College of Art, Zhejiang Normal University, Jinhua 321004, China; dolly1395105560@gmail.com; 2 College of Life Sciences, Zhejiang University, Hangzhou 310058, China; lmiaomiao02@163.com (M.L.); 22307024@zju.edu.cn (M.Z.)

**Keywords:** in situ hybridization, spatial transcriptomics, MERFISH, RAEFISH, HCR-FISH, RNAscope, cell segmentation, single-molecule imaging, spatial multi-omics

## Abstract

In situ hybridization (ISH) has undergone a rapid evolution from a low-throughput histological staining technique to a diverse family of modern methods for sensitive, specific and multiplexed molecular detection in intact cells and tissues, and to a cornerstone technology for image-based spatial transcriptomics. This transformation has been driven by advances in probe design, signal amplification, cyclic imaging, combinatorial barcoding, automated fluidics and computational decoding, which together allow RNA molecules to be measured within preserved cellular and tissue architecture. In this review, we examine the molecular and engineering principles that underlie modern ISH methods and their extension into ISH-based spatial profiling, with emphasis on hybridization chain reaction, branched-DNA amplification, SABER-FISH, rolling-circle-amplification-based approaches, seqFISH, MERFISH, RAEFISH and selected commercial implementations. We discuss how sensitivity, specificity, tissue compatibility, optical crowding, imaging burden, cost, reproducibility and computational uncertainty shape the practical use of each method. Sequencing-based spatial capture platforms are not reviewed comprehensively, but are considered where comparative benchmarks help clarify trade-offs in spatial resolution, transcriptome breadth, tissue area or analytical interpretation. We also consider how recent benchmarking and standardization efforts are beginning to define quantitative criteria for comparing platforms, and how advances in segmentation, barcode decoding, spatial integration and cell–cell communication analysis convert raw images into biological insight. Finally, we highlight applications in targeted transcript detection, tissue-based validation, neuroscience, cancer, developmental biology, non-model organisms and spatial functional genomics, where modern ISH methods and ISH-based spatial profiling provide information that bulk and dissociated single-cell approaches cannot capture. Together, these developments trace how ISH has expanded from targeted molecular visualization into a broad methodological framework for in situ detection and spatially resolved transcriptomic analysis.

## 1. Introduction

Molecular biology has long focused on identifying which genes a cell expresses and how much of each transcript it produces. It is now clear, however, that where transcripts are located—within cells, across tissue compartments and relative to neighboring cells—can be equally informative. Spatial gene expression shapes developmental patterning, immune surveillance, tissue repair and the ecological niches that sustain or constrain tumor growth. Bulk and single-cell RNA sequencing have transformed transcriptomics, but they usually require tissue dissociation or homogenization and therefore lose the spatial relationships that give gene expression its anatomical and functional context.

In situ hybridization (ISH), first demonstrated by Gall and Pardue in 1969, provided one of the earliest routes to sequence-specific molecular visualization in intact specimens [1]. The principle is simple: a labeled nucleic-acid probe hybridizes to a complementary DNA or RNA target within fixed cells or tissue sections. By preserving tissue architecture while revealing the location of specific nucleic acid species, ISH links molecular identity directly to histological context. For decades, this made ISH indispensable for validating gene-expression patterns, localizing candidate transcripts, mapping developmental markers and detecting pathogens or biomarkers in tissues. Early implementations relied on radioactive or chromogenic detection and were usually applied to small numbers of targets, but the conceptual strength of ISH was already clear: it could answer not only whether a molecule was present, but where it was positioned in a cell or tissue.

Modern ISH has expanded this principle far beyond classical probe staining. Advances in oligonucleotide probe design, locked-nucleic-acid and peptide-nucleic-acid chemistries, signal amplification, fluorescence imaging, cyclic readout and automated fluidics have increased the sensitivity, specificity, robustness and multiplexing capacity of ISH. Single-molecule RNA fluorescence in situ hybridization (smFISH) made individual transcript counting possible at cellular and subcellular resolution [2,3]. Hybridization chain reaction, branched-DNA amplification, SABER-FISH and rolling-circle-amplification-based strategies further diversified the ways in which weak molecular recognition events can be converted into detectable signal. These methods remain valuable in their own right for researchers who need targeted transcript detection, whole-mount imaging, FFPE-compatible assays, non-model organism workflows, low-abundance RNA detection or moderate-plex validation.

At the same time, these molecular innovations have become the foundation of image-based spatial transcriptomics. Combinatorial barcoding, cyclic imaging, error-correcting codebooks, rolling-circle amplicons and computational decoding have transformed ISH-derived assays from candidate-gene localization into high-plex spatial profiling platforms such as seqFISH, MERFISH, RAEFISH and several commercial implementations. The shift is therefore twofold. Modern ISH provides a toolkit for more sensitive, specific and multiplexed in situ detection; it also supplies the molecular logic from which many spatial transcriptomics technologies have been engineered. For this reason, understanding modern ISH chemistry is essential not only for choosing a targeted assay, but also for interpreting the strengths and limitations of high-plex spatial transcriptomic data.

This review follows the methodological evolution of ISH from molecular chemistry to spatial-transcriptomic implementation. We first examine modern ISH methods as practical molecular tools, focusing on probe design, amplification architecture, sample compatibility, multiplexing strategy and common sources of experimental failure. We then discuss how these principles are extended into high-plex spatial profiling through cyclic imaging, combinatorial barcoding, RCA-based readout, commercial platform engineering and computational decoding. Sequencing-based spatial capture technologies, including array-based, bead-based and slide-based transcript-capture methods, have been reviewed extensively elsewhere and are not covered comprehensively here [4,5,6,7]; they are discussed only where comparative benchmarks help clarify trade-offs in spatial resolution, transcriptome breadth, tissue area or analytical interpretation. The following sections therefore move from the molecular and engineering foundations of modern ISH and ISH-derived high-plex platforms, to the computational steps that convert microscopy images into spatially resolved molecular measurements, and finally to applications in neuroscience, cancer, developmental biology, non-model organisms and spatial functional genomics. Together, these sections trace how ISH has developed from targeted molecular visualization into a diverse set of methods for sensitive in situ detection and spatially resolved transcriptomic analysis.

## 2. Core Molecular Foundations: Sensitivity, Specificity, and Signal

At its core, in situ hybridization exploits the thermodynamic stability of Watson–Crick base pairing to localize specific nucleic acid sequences within morphologically preserved cells and tissues. A labeled probe—a single-stranded DNA or RNA molecule complementary to the target—is introduced into a fixed and permeabilized specimen, where it diffuses through the cellular matrix and hybridizes to its cognate sequence. The specificity of this recognition event depends on probe length, GC content, formamide concentration, and hybridization temperature, all of which together define the stringency window that distinguishes true target binding from off-target cross-hybridization. Once hybridized, the probe–target duplex is detected through a reporter system: historically a radioactive isotope or enzyme-linked chromogen, and in modern protocols a fluorophore or a molecular scaffold that recruits fluorescent labels. The central challenge in ISH is to achieve both specific target recognition and sufficient signal for reliable detection, without compromising spatial resolution. These two problems—probe engineering and signal amplification—have driven the molecular innovations.

### 2.1. Probe Engineering

#### 2.1.1. Oligonucleotide Probe Pools Versus Long-Chain Probes

**Figure 1:**
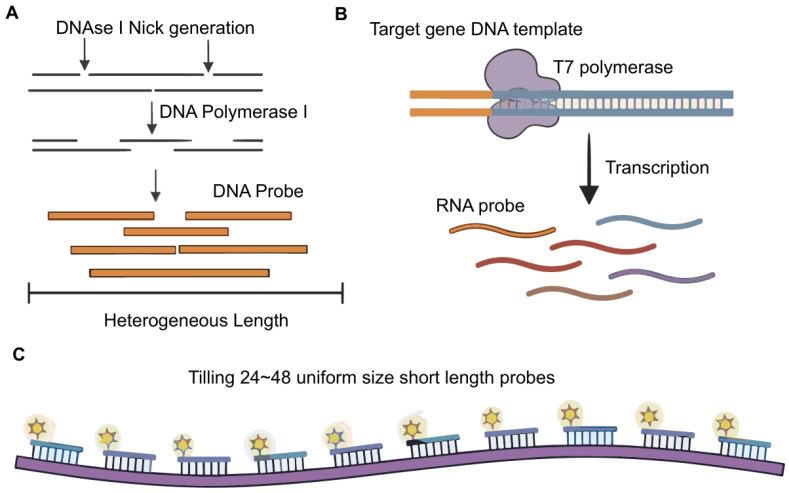
Probe-generation strategies for in situ hybridization. (**A**) Nick translation generates labeled DNA probes by combining DNase I-mediated nicking with DNA polymerase I-dependent nucleotide replacement, producing probe fragments of heterogeneous lengths. (**B**) In vitro transcription using T7 RNA polymerase produces complementary RNA probes from a DNA template. (**C**) Modern oligonucleotide probe pools consist of 24–48 short, uniformly designed probes tiled across the same transcript, enabling sensitive detection, precise probe composition, and compatibility with highly multiplexed imaging workflows.

The sensitivity and specificity of any ISH experiment are fundamentally determined by the probe. Classical long probes, typically generated by nick translation or in vitro transcription, often provided strong hybridization signals but offered limited flexibility for multiplexing. Their greater length and sequence complexity could also increase the risk of cross-hybridization, particularly when repetitive or homologous regions were not carefully excluded. The modern paradigm centers on pools of short synthetic oligonucleotides (typically 20–50 nucleotides), each targeting a different subsequence of the same mRNA. By tiling 24–48 oligos per transcript, smFISH achieves single-molecule sensitivity through the statistical accumulation of individually dim but collectively bright fluorescent puncta (Figure 1C) [2,3]. This oligo-pool strategy is the foundation of virtually every high-plex ISH method in use today, including MERFISH, seqFISH, and the Xenium and CosMx commercial platforms. A critical practical advantage of oligo pools is their compatibility with array-based synthesis, and several software pipelines support genome-scale oligo probe design (for example, OligoMiner [8]). Complex libraries targeting thousands of genes can be generated from a single pooled oligonucleotide synthesis and then PCR-amplified to produce sufficient material for downstream probe preparation at low per-experiment cost.

#### 2.1.2. Advanced Chemistries: Locked Nucleic Acids and Peptide Nucleic Acids

Chemical modifications to the probe backbone or sugar moiety can enhance binding affinity, mismatch discrimination and nuclease resistance. Locked nucleic acids (LNAs), in which a methylene bridge constrains the ribose into a C3′-endo conformation, increase duplex stability and improve single-nucleotide mismatch discrimination [9]. LNA-modified probes are particularly valuable for detecting short targets such as microRNAs, where high affinity permits stringent washing without a major loss of sensitivity [10]. Peptide nucleic acids (PNAs), in which the sugar-phosphate backbone is replaced by N-(2-aminoethyl) glycine units, hybridize to complementary nucleic acids with high affinity and resist nuclease and protease degradation [11]. These chemistries are useful when short targets, sequence discrimination or sample degradation are limiting, although their higher cost and narrower design space make them less central to large array-synthesized oligonucleotide pools than standard DNA probes. PNA-FISH remains widely used for telomere length measurement and related cytogenetic applications [12].

### 2.2. Signal Amplification Architectures

Probe design determines what can be detected; signal amplification determines whether it can be seen. A single fluorophore conjugated to a single oligonucleotide probe often produces a signal that is difficult to distinguish from cellular autofluorescence, particularly in tissue types rich in lipofuscin, collagen or chlorophyll. The smFISH strategy addresses this by tiling dozens of singly labeled probes along each transcript, so that the aggregate signal from a bound probe set exceeds the background even though any individual probe does not (Figure 1C) [2,3]. This approach is most straightforward for longer transcripts that can accommodate many non-overlapping oligonucleotides, but the practical lower boundary depends on probe density, target abundance, background, imaging conditions. Enzymatic and non-enzymatic amplification architectures address this constraint by converting each probe-target binding event into a localized cascade of reporter molecules, including branching oligonucleotide trees, self-assembling hairpin polymers and clonally amplified DNA nanoballs. These strategies differ in whether they require enzymes, how they suppress background in the absence of target, how well they preserve spatial localization and how readily they can be integrated into multiplexed workflows (Table 1).

Viewed together, these amplification schemes represent different solutions to the same measurement problem. HCR is often attractive for whole-mount or non-model validation because target-dependent initiator assembly reduces background from isolated probe binding. RNAscope is particularly strong for robust low-to-moderate plex assays in FFPE material. SABER is useful when signal strength must be tuned through programmable concatemers, whereas RCA and padlock-based approaches are well suited to digital amplicon counting and barcode decoding.

#### 2.2.1. Hybridization Chain Reaction (HCR)

**Figure 2:**
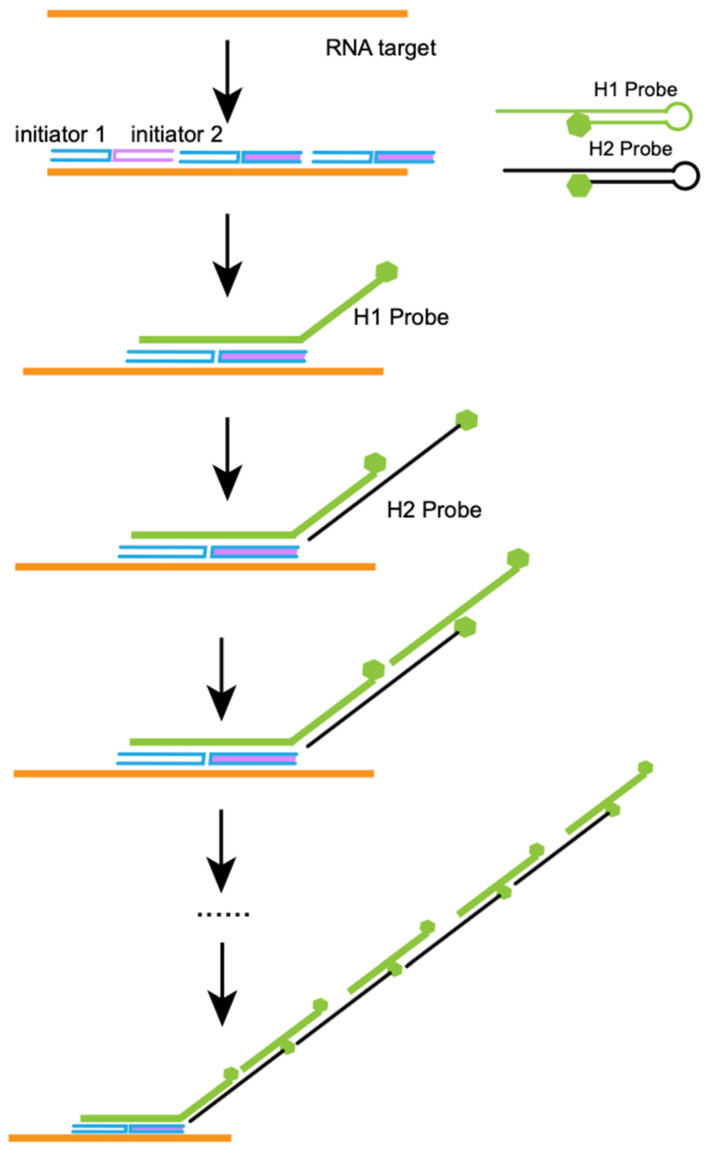
Mechanism of HCR-FISH signal amplification. Target RNA is first recognized by a pair of adjacent initiator probes. Each initiator probe hybridizes to a neighboring region of the RNA target and contributes part of the HCR initiation sequence. Only when the two initiators bind in close proximity is a functional initiator complex formed, allowing signal amplification to begin. The initiator first hybridizes to the exposed input domain of the metastable H1 hairpin probe, opening H1 through strand displacement and exposing a new single-stranded output domain. This exposed domain then anneals to the input domain of the H2 hairpin probe, opening H2 and adding it to the growing amplification polymer. Opening of H2 exposes a sequence equivalent to the H1-opening initiator, which recruits and opens another H1 probe. Repeated alternating H1 and H2 opening and annealing generates a long, tethered DNA polymer decorated with fluorophores, thereby producing a strong localized fluorescent signal at the RNA target. Because untriggered H1 and H2 hairpins remain kinetically trapped, signal amplification occurs only at sites where the initiator probes have correctly assembled on the target RNA, providing the intrinsic background suppression that distinguishes HCR from enzyme-dependent amplification chemistries.

Hybridization chain reaction (HCR) amplifies signal using two fluorophore-labeled DNA hairpins, H1 and H2, that are designed to remain folded and inert until triggered [13]. In HCR v3.0, adjacent binding of two split-initiator probes on the target mRNA reconstructs a full initiator sequence. This initiator opens H1 through toehold-mediated strand displacement, exposing a sequence that opens H2. The opened H2 then exposes a sequence that opens another H1, and the cycle repeats, producing a localized fluorescent polymer at the target site (Figure 2). Because either probe alone carries only half of the initiator, nonspecific binding does not efficiently trigger polymerization, thereby suppressing background [14]. HCR v3.0 supports up to five orthogonal amplifier channels, enabling simultaneous detection of five targets in a single hybridization round, and has been validated across a broad range of embryos, tissues, and whole-mount preparations [14]. More recent extensions have adapted HCR for stronger fluorescence or catalytic readouts. HCR-Cat combines target-triggered HCR with hapten-labeled amplification polymers followed by antibody-mediated horseradish peroxidase (HRP) catalysis and tyramide signal amplification, thereby greatly increasing signal for short or low-abundance targets under the tested conditions [15]. The same study introduced related immunological and multi-step HCR variants, including HCR-Immuno and HCR-Multi [15]. These approaches illustrate how HCR can be coupled to secondary amplification chemistries, although their broad performance across tissue types and laboratories remains less established than that of HCR v3.0.

HCR v3.0 has become one of the most widely used signal-amplification chemistries for targeted ISH, in part because the split-initiator design provides intrinsic background suppression without requiring enzymatic processing of the tissue. This property has made the method especially useful in whole-mount embryos, plant tissues and other specimens in which enzymatic protocols are difficult to control or in which autofluorescence and tissue depth confound conventional fluorescent probes. For low-abundance targets, performance still depends on probe penetration, hairpin quality and background from fixation chemistry, which is part of the motivation for the brighter or catalytic extensions described above.

#### 2.2.2. Branched DNA Systems

Branched DNA–style signal amplification increases detection sensitivity by building a hierarchical oligonucleotide scaffold on top of a target-bound probe. In these systems, target recognition first creates a binding site for a preamplifier, which then recruits multiple amplifier molecules, each of which in turn binds multiple labeled reporter probes. This tree-like assembly converts a single hybridization event into a large, localized fluorescent or chromogenic signal while helping preserve spatial precision.

**Figure 3:**
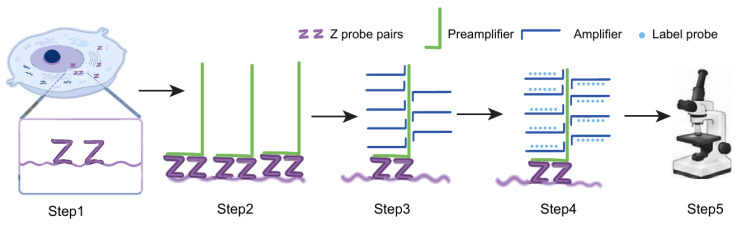
Working principle of ACDscope/RNAscope signal amplification. (Step 1), fixed and permeabilized cells or tissue sections preserve target RNA molecules in situ, allowing target-specific Z probe pairs to access intracellular RNA. (Step 2), multiple Z probe pairs hybridize along the same target RNA molecule. Each Z probe pair consists of two adjacent probes that bind contiguously to the target sequence; only when both probes are correctly positioned does their combined tail region form a docking site for the preamplifier. (Step 3), a preamplifier molecule binds to the docking site created by one valid Z probe pair. (Step 4), multiple amplifier molecules bind to the preamplifier, and multiple label probes subsequently bind to each amplifier, generating a branched signal amplification structure. For clarity, (Steps 3,4) illustrate signal amplification from a single Z probe pair, although multiple Z probe pairs can be tiled along one target RNA to increase detection sensitivity. (Step 5), accumulated fluorescent or chromogenic labels are visualized microscopically as discrete puncta, revealing the spatial distribution of target RNA molecules within cells or tissues.

A widely used implementation of this strategy is RNAscope, a commercial RNA ISH platform that combines a proprietary double-Z probe design with a branched amplification cascade [16]. In RNAscope, target-specific Z-probes bind in pairs to the mRNA; the paired probes together create a binding site for a pre-amplifier oligonucleotide, which in turn recruits multiple amplifier oligos, each bearing 20 or more fluorescent label probes (Figure 3). This hierarchical assembly was reported to generate approximately 400-fold to 8000-fold signal amplification per mRNA molecule while maintaining single-molecule sensitivity in tissue sections [16]. The paired-probe requirement and strong amplification have made RNAscope particularly valuable for formalin-fixed, paraffin-embedded (FFPE) material, the dominant format of clinical pathology archives [17,18]. At the same time, its proprietary probe design, limited panel size relative to high-plex imaging platforms, potential signal saturation for highly expressed genes and reagent cost make it better suited to robust targeted assays than to open-ended transcriptome-scale discovery.

#### 2.2.3. SABER-FISH and Primer Exchange Reaction

**Figure 4:**
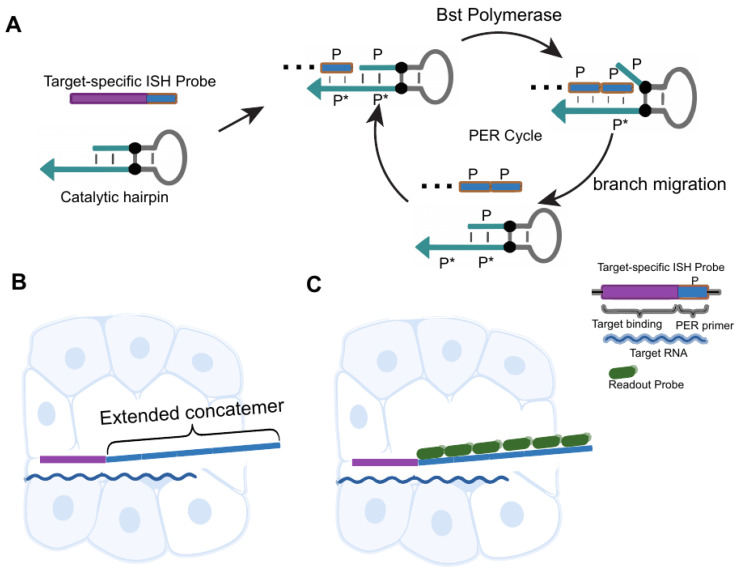
PER-mediated concatemerization enables SABER-FISH signal amplification. (**A**) Target-specific ISH probes are synthesized with a 3′ PER primer and extended in vitro by primer exchange reaction (PER). In each PER cycle, the primer transiently hybridizes to a catalytic hairpin through the P/P* domain and is extended by Bst LF polymerase, a strand-displacing DNA polymerase. The hairpin is designed so that each cycle adds one P domain. In the SABER/PER design, the concatemer sequence excludes guanine bases, and G/C-containing positions in the hairpin act as polymerase terminators because dGTP is omitted from the reaction. This prevents polymerase read-through beyond the intended P-domain template. After extension, branch migration displaces the elongated primer and restores the catalytic hairpin, allowing repeated cycles to generate a single-stranded concatemer composed of tandem P domains. (**B**) In fixed cells or tissue, only the target-binding region of the extended ISH probe hybridizes to the target RNA, while the PER-generated concatemer remains single-stranded and projects away from the target. (**C**) Fluorescent readout probes complementary to the repeated P domains hybridize along the concatemer. Because each P domain can recruit one fluorescent readout probe, each target-bound ISH probe accumulates multiple fluorophores, producing amplified fluorescence signal for imaging.

Signal Amplification by Exchange Reaction (SABER) separates target recognition from signal generation [19]. First, a gene-specific oligonucleotide probe hybridizes to the target RNA or DNA in the sample. Rather than carrying only a single fluorophore, this probe is coupled to a short DNA primer sequence that can be extended in vitro before the hybridization experiment. Using the Primer Exchange Reaction (PER), this primer is converted into a long single-stranded DNA concatemer containing many repeated sequence units [19,20]. PER itself is an isothermal extension reaction programmed by a catalytic DNA hairpin template and driven by a strand-displacing polymerase. In each cycle, the primer binds the hairpin template, a short DNA sequence is copied onto the primer, and the extended strand is released so that the process can repeat. Repeated cycling appends the same sequence motif many times, generating a concatemer of defined composition and tunable length [20]. After the concatemer-bearing probe hybridizes to its target in the specimen, the concatemer functions as an amplification scaffold. Multiple fluorescent imager oligonucleotides, each complementary to the repeated concatemer sequence, can bind along the same scaffold. Thus, a single target-binding event is converted into the recruitment of many fluorophores, producing a much brighter signal than conventional single-fluorophore probe designs (Figure 4) [19]. A further advantage of SABER is that signal amplification is decoupled from probe hybridization. Signal strength can be tuned by changing concatemer length or by adding branched amplifier layers, and different orthogonal concatemer sequences can be assigned to different targets for multiplexed imaging. Because the fluorescent imagers are short oligonucleotides that can be stripped and replaced, the same concatemer scaffold can also be re-imaged in successive rounds, enabling exchange-based multiplexing without re-hybridizing the original gene-specific probes [19].

An attractive feature of SABER is that the amplification scaffold can be tuned independently of the gene-specific probe, so that the same probe library can be re-imaged at different brightness levels or with different barcode assignments. This flexibility comes at the cost of substantial probe-synthesis and quality-control effort, since concatemer length, branch architecture and stripping efficiency directly shape signal-to-noise and limit how many imaging rounds can be accumulated before barcode decoding becomes unreliable.

#### 2.2.4. Rolling Circle Amplification (RCA)-Based FISH

RCA-based FISH methods convert each target molecule into a localized DNA amplicon that can be visualized by sequencing or hybridization readout [21,22]. In a typical workflow, the target mRNA is first reverse-transcribed into cDNA within the fixed specimen. A padlock probe is then designed so that its two ends hybridize to adjacent sequences on the cDNA. When both ends align correctly, they are ligated to form a closed circular DNA molecule, thereby ensuring that amplification occurs only on the correctly recognized target [21]. This circularized padlock probe then serves as the template for rolling circle amplification. A strand-displacing DNA polymerase extends from a primer bound to the circle and repeatedly copies around the circular template many times without dissociating. Because the same short circle is copied over and over, the reaction generates a long single-stranded DNA concatemer containing many tandem repeats of the padlock sequence. Importantly, this product remains tethered near the original target site, where it collapses into a compact, bright submicron amplicon, often termed a rolony [21,22].

The critical advantage of RCA is that a single target-recognition event is converted into many local copies of the same DNA sequence. This repeated amplicon can then be decoded either by in situ sequencing, using sequencing-by-ligation or sequencing-by-synthesis, or by iterative FISH-based readout, in which fluorescent probes hybridize to barcode sequences embedded in the rolony. In this sense, the rolony functions as a localized molecular barcode amplifier rather than merely as a brighter conventional probe signal [21,22]. Because each successfully ligated padlock probe ideally gives rise to one amplified rolony, RCA-based methods are often described as digitally countable: each punctum corresponds to an individual molecular detection event. Digital counting does not, however, equal complete capture, since the upstream reverse transcription and padlock ligation steps each carry their own efficiency, and dense subcellular regions or highly expressed genes can produce overlapping amplicons that challenge spot calling. The same principle underlies a range of in situ sequencing and imaging platforms, including the padlock-probe chemistry used in Xenium. More recently, RAEFISH has retained the sensitivity and digital nature of RCA while redesigning the probe architecture to remain compatible with pooled oligonucleotide synthesis, thereby making transcriptome-scale image-based profiling more practical [22,23].

The same RCA logic also underlies several sequencing-like in situ readouts. STARmap and STARmap PLUS combine in situ amplification with hydrogel-based tissue chemistry and sequencing-style decoding, extending transcriptomic imaging into three-dimensional or histology-integrated brain mapping [22,24]. HybISS similarly uses padlock-probe recognition, RCA and hybridization-based decoding to visualize targeted transcripts in tissue sections [25]. Taken together, these methods illustrate the broader role of RCA in spatial transcriptomics as a strategy for converting weak molecular recognition events into brighter, sequence-encoded amplicons, while sharing a common set of upstream dependencies on reverse-transcription efficiency, ligation, amplicon density and registration across imaging cycles.

## 3. Engineering the High-Plex Frontier

### 3.1. Signal Management for Sequential Rounds

High-plex ISH methods aim to measure large numbers of transcripts within the same specimen while preserving spatial resolution. Because the number of spectrally separable fluorophores that can be imaged simultaneously is limited, many high-plex platforms achieve scalability by distributing transcript identity across multiple rounds of hybridization and imaging, using either sequential probing or combinatorial barcoding strategies. In these workflows, the fluorescent signal from one imaging round must be efficiently removed before the next round of readout probe hybridization can begin, and each additional cycle creates opportunities for tissue drift, photobleaching, incomplete stripping and accumulated barcode bit errors. The achievable plex of a barcoding scheme is therefore inseparable from registration accuracy, signal-removal efficiency and per-round quality control.

In practice, signal removal is typically achieved either by chemically stripping short readout probes—for example, with formamide-based washes in MERFISH and related workflows—or by using engineered cleavage chemistries built into decoder probes or amplification scaffolds [15,26,27,28]. MERFISH commonly uses short readout probes with relatively low thermal stability, enabling rapid formamide-based removal between imaging rounds, often on the order of minutes. Efficient signal removal is essential for maintaining barcode fidelity across the dozens of imaging rounds required for transcriptome-scale experiments.

### 3.2. Multiplexing Strategies

#### 3.2.1. Sequential ISH and Temporal Barcoding

**Figure 5:**
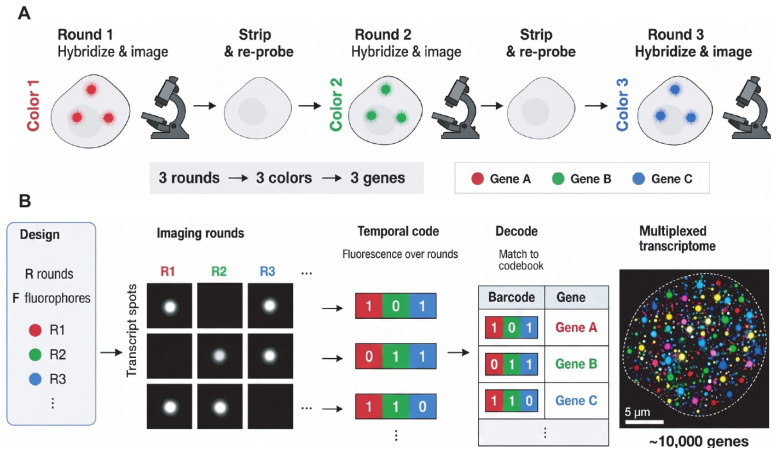
Sequential ISH and combinatorial barcoding for multiplexed RNA imaging. (**A**) Sequential ISH. Targets are detected through iterative rounds of probe hybridization and imaging, with signal stripping and re-probing between rounds. In this simplified example, a different fluorophore is used in each round to visualize target transcripts. The labels Gene A, Gene B and Gene C are shown for illustration only; in practice, each imaging round can assay multiple genes rather than a single target. Thus, in the basic sequential format, the number of detectable genes increases approximately linearly with the number of imaging rounds. (**B**) Combinatorial temporal barcoding in seqFISH/MERFISH. Transcript identity is encoded by the sequence of fluorescence signals observed across successive imaging rounds. A transcript spot is imaged across multiple rounds, generating a temporal barcode that is matched to a predefined codebook for gene assignment. In this strategy, the coding capacity expands combinatorially with the number of fluorophores (F) and imaging rounds (R), such that, in principle, up to F^R barcodes can be generated. This enables highly multiplexed transcriptome-scale imaging, illustrated here by detection of approximately 10,000 genes within a single experiment.

The simplest route to multiplexing is to perform sequential rounds of hybridization and imaging, in which one or a small number of targets are detected per round and the sample is subsequently stripped and re-probed [26]. In this basic format, the number of detectable genes increases approximately linearly with the number of imaging rounds (Figure 5A). More advanced implementations use temporal or combinatorial barcoding schemes to expand coding capacity beyond the number of fluorophores that can be resolved in a single round. In seqFISH, transcript identity is decoded from the sequence of fluorescence signals observed across successive rounds, such that, in principle, F fluorophores imaged over R rounds can encode up to F^R barcodes (Figure 5B) [26,28]. seqFISH+ greatly expanded this strategy to transcriptome-scale imaging of approximately 10,000 genes using 60 rounds of imaging in three pseudocolor channels, and later extensions integrated the mapping of over 100,000 genomic loci, nascent transcripts from approximately 18,000 genes, and subnuclear structural features in single nuclei [28,29]. The strength of sequential schemes is conceptual simplicity, but the cost is that imaging time, reagent use and cumulative technical variation scale linearly with the number of probed genes, which is one reason why transcriptome-scale studies generally adopt compressed barcoding schemes such as those described in the next section.

#### 3.2.2. Combinatorial Barcoding: MERFISH and Error Correction

Multiplexed Error-Robust Fluorescence In Situ Hybridization (MERFISH), developed by the Zhuang laboratory [27,30,31], introduced the use of error-robust binary barcodes for massively parallel transcript identification. Each gene is assigned a unique binary codeword drawn from a codebook with Hamming distance constraints, such that single-bit errors arising from missed or spurious fluorescent detections can be detected and corrected. In a typical implementation, each transcript is labeled with encoding probes bearing overhang sequences; in successive imaging rounds, dye-labeled readout probes hybridize to specific overhangs, producing a binary on/off pattern that constitutes the barcode. Hamming distance 4 codes enable single-error correction and double-error detection, and this molecular error-correction strategy is what allows MERFISH to scale to transcriptome size without an explosion of misassigned reads.

MERFISH has been progressively scaled from initial demonstrations of approximately 1000 genes to implementations profiling over 10,000 genes with high detection efficiency and subcellular RNA compartmentalization analysis. The misidentification rates reported in landmark MERFISH demonstrations, often below 4%, are achieved with sparse codebooks such as 4-on-of-94 schemes, which reduce optical crowding and provide the redundancy required for error correction; denser codebooks recover more bits per round but can also increase decoding ambiguity [27,30,31]. The physical limit of combinatorial barcoding is therefore optical crowding: as the number of targeted species increases, the density of fluorescent spots per imaging field rises, and eventually spot overlap, chromatic aberration and residual signal from earlier rounds combine to degrade decoding accuracy. This is one reason why reported MERFISH performance varies with panel design, tissue type and decoding pipeline as well as with the underlying chemistry.

#### 3.2.3. RAEFISH: Transcriptome-Scale ISH Without Sequencing

Reverse-padlock Amplicon Encoding Fluorescence In Situ Hybridization (RAEFISH), reported in 2025 and discussed in 2026 as a notable advance in the field, extends image-based ISH toward transcriptome-scale targeting [23,32]. Mechanistically, RAEFISH uses a reverse-padlock design in which pooled gene-specific probes first prime cDNA generation; a splint-assisted ligation step then circularizes the padlock-like template, and rolling circle amplification converts each captured transcript into a bright amplicon that is decoded by MERFISH-style combinatorial readout imaging. This architecture addresses a practical bottleneck of earlier RCA-based approaches by keeping large probe libraries compatible with pooled oligonucleotide amplification rather than relying only on individually synthesized padlocks.

In the initial demonstration, RAEFISH used a probe set spanning approximately 23,000 human genes and detected, on average, 3749 RNA molecules from 1287 distinct genes per A549 cell, with a probe-library cost of approximately $158 [23]. These figures represent a substantive step toward transcriptome-scale imaging, and they also help clarify what “whole-transcriptome” currently means in this context: the probe library covers the transcriptome, but only a subset of genes is recovered in any individual cell, and the headline cost reflects probe synthesis and amortization rather than the full experimental cost of instrument time, imaging cycles, decoding compute and labor.

RAEFISH measurements in A549 cells showed bulk RNA-seq concordance (r, 0.66) and inter-replicate reproducibility (r, 0.85), and mouse liver experiments identified major cell types and periportal-pericentral hepatocyte zonation [23]. How RAEFISH compares with MERFISH and seqFISH at matched panel size remains an open question that depends on shared tissues, fixation conditions and analysis pipelines. The same study introduced Perturb-RAEFISH, in which guide RNA (gRNA) sequences from a CRISPR perturbation library are imaged in situ and linked to spatial transcriptomic phenotypes; together with Perturb-FISH and the spatial CRISPR genomics work of Dhainaut et al. [33], this defines an emerging class of image-based perturbation screens that differ in panel scale, perturbation readout and tissue model.

### 3.3. Microfluidic Integration and Automation

The scalability of high-plex ISH depends critically on the automation of fluidic handling. Each imaging round in a MERFISH or seqFISH experiment involves multiple precise steps: readout probe hybridization, wash, imaging, stripping, and re-wash. Manual execution of 50–100 such cycles is impractical and introduces variability. Modern ISH platforms integrate programmable fluidic systems that deliver microliter-scale reagent volumes through flow cells or over tissue sections with minimal dead volume. Fluorinated oils such as FC-40 serve as immiscible barriers that prevent tissue dehydration during long imaging sessions and reduce reagent consumption by eliminating aqueous evaporation. Surfactant engineering at the oil–aqueous interface maintains tissue wettability without disrupting probe hybridization.

Open-source microfluidic implementations developed around MERFISH- and seqFISH-style workflows have helped demonstrate that automated cyclic hybridization and imaging can be achieved without fully proprietary instrumentation [5,27,28]. However, these systems still demand substantial expertise in hardware integration, calibration, fluidic control, and long-run maintenance, which remains one reason commercial platforms continue to offer a practical reliability advantage for many laboratories.

Automation also highlights a broader limitation of cyclic image-based ISH: throughput is constrained not only by barcode capacity, but by the ability of the tissue and molecular signal to survive repeated rounds of chemistry and imaging. Each cycle requires probe delivery, hybridization, washing, signal removal and image acquisition; over many cycles, these steps can increase imaging time, promote sample drift or deformation, reduce RNA or signal retention, and make cross-round registration more difficult. These constraints become especially important for large tissue areas, thick specimens and highly autofluorescent samples, where direct imaging in tissue may require extensive z-sectioning or background correction. EEL FISH illustrates a complementary strategy for addressing this problem. Instead of repeatedly imaging RNA molecules within the tissue section itself, EEL FISH electrophoretically transfers RNA from the tissue onto a capture surface, allowing cyclic FISH readout on a flatter and less autofluorescent substrate [34]. This design reduces the amount of imaging required, supports large-area single-cell profiling and can improve analysis of challenging samples such as human brain tissue by removing autofluorescent lipofuscin. The trade-off is that spatial accuracy now depends on efficient vertical RNA transfer and preservation of registration between the original tissue and the capture surface.

### 3.4. Commercial Platforms and Their Molecular Engineering

Commercial imaging-based spatial transcriptomics platforms translate the core molecular principles of high-plex ISH into standardized and automated workflows. They differ in chemistry, for example padlock probe plus RCA and iterative FISH in Xenium, smFISH- or MERFISH-derived barcoding strategies in CosMx and MERSCOPE, cyclic single-molecule FISH in Molecular Cartography (Resolve Biosciences), and padlock/RCA-based cyclic sequencing on G4X, but share the goal of localizing many RNA molecules, and in some workflows proteins, within intact tissue at single-cell to subcellular resolution. Three caveats are important when reading the descriptions and numbers that follow. First, panels, chemistries and software versions are revised on a timescale of months, so any quantitative specification is a snapshot of a particular platform release rather than a fixed instrument property. Second, the available evidence is heterogeneous: peer-reviewed independent benchmarks, peer-reviewed studies from the platform-developer laboratory, vendor specifications and early-access or conference reports each carry different weight, and we therefore identify the type of evidence supporting each claim. Third, results from these platforms are sensitive to tissue type, panel design, segmentation choices and analysis workflow, so any single performance metric should be read as conditional on the cited experimental context.

Independent benchmarking has begun to reveal how strongly platform performance depends on tissue, panel design and analysis workflow. Wang et al. [35] benchmarked Xenium, MERSCOPE and CosMx across tumor and normal tissue microarrays (TMAs) and reported that Xenium generated higher transcript counts per matched gene without an apparent specificity penalty, while Xenium and CosMx showed stronger concordance with orthogonal single-cell transcriptomics. Ozirmak Lermi et al. [36] compared CosMx, MERFISH and Xenium on FFPE lung adenocarcinoma and pleural mesothelioma, reporting platform-specific differences in false-discovery estimates, cell filtering, segmentation behavior and concordance with DSP WTA. Ren et al. [37] and Cervilla et al. [38] broadened the comparison to include sequencing-based technologies as well as imaging-based platforms, emphasizing that platform ranking can vary by tissue type, metric, panel design, section quality and analysis workflow. These studies argue against a single universal hierarchy of instruments; platform choice is better viewed as a specimen- and question-specific optimization problem (Table 2).

## 4. The Computational Bridge: From Images to Spatial Biology

### 4.1. AI-Driven Image Analysis

#### 4.1.1. Cell Segmentation: Foundation Models

Accurate cell segmentation is an essential first step in extracting single-cell expression profiles from image-based spatial transcriptomics data. A key recent development is the use of foundation-model-inspired approaches that aim to generalize across cell types, imaging modalities and tissue architectures. CellSAM integrates a Vision Transformer encoder with the Segment Anything Model (SAM) mask decoder and uses a learned object detector, CellFinder, to generate bounding-box prompts automatically [39]. Trained on diverse imaging data spanning mammalian tissues, yeast and bacteria across fluorescence, brightfield and phase-contrast modalities, CellSAM has shown broad generalization and has been applied to spatial transcriptomics images, including MERFISH and seqFISH datasets, within the Polaris spatial-analysis workflow.

A closely related development is Cellpose-SAM, reported as a 2025 preprint, which adapts a SAM-derived transformer backbone to the Cellpose gradient-flow framework [40]. Its practical value lies in improved out-of-distribution performance across common imaging perturbations, including channel shuffling, cell-size variation, shot noise, downsampling and blur, while retaining compatibility with the broader Cellpose ecosystem [41] for fine-tuning, human-in-the-loop training, image restoration and three-dimensional segmentation. Commercial spatial-transcriptomics platforms have also invested heavily in platform-specific segmentation workflows, often building on Cellpose- or DeepCell-style architectures [41,42] that combine nuclear, cytoplasmic, membrane and/or morphology channels; however, the exact algorithms and performance characteristics are version- and assay-dependent.

Even with these advances, segmentation remains a major source of single-cell-level variability in ISH-based spatial transcriptomics. Boundary calls are sensitive to staining choice, tissue quality, cell-packing density and the availability of membrane or cytoplasmic signal. Errors in segmentation propagate directly into molecule-to-cell assignment, cell-type classification, neighborhood analysis and cell-cell communication inference, particularly for densely packed tissues, cells with weak membrane signal or samples with unusual morphology. Thus, foundation models improve generalization, but they do not remove the need for dataset-specific quality control and, in many cases, manual inspection or targeted model refinement.

#### 4.1.2. Spot Calling and Barcode Decoding

The decoding of combinatorial barcodes from raw fluorescence images requires algorithms that can resolve sub-diffraction-limit spots, assign them to genes and estimate confidence in each assignment. Probabilistic approaches model each observed fluorescent punctum as a mixture of true molecular signal and structured noise sources such as autofluorescence, optical crosstalk and incomplete stripping, rather than relying solely on hard thresholding [43]. Maximum-likelihood and Bayesian decoding frameworks then estimate the most probable barcode for each detected spot from the observed intensity pattern across imaging rounds and can report explicit confidence scores for downstream filtering [43]. Quality control tooling for imaging-based spatial transcriptomics is moving toward auditable. For example, the Spatial Touchstone initiative introduced SpatialQM as a standardized metric suite for cross-site and cross-platform evaluation [44]. Complementary pipelines such as SpatialQC provide end-to-end QC reporting for spatial transcriptome datasets (Mao et al., 2024), and SpotSweeper demonstrates how dataset-level artifacts and decode-level errors can be detected and corrected in MERFISH-style data [45]. Quality control metrics such as per-spot decoding confidence, per-gene false discovery, blank-probe behavior, spatial consistency and cross-run reproducibility; standardized initiatives are discussed below in Section 4.4.

### 4.2. Spatial Data Integration with scRNA-seq

#### 4.2.1. Probabilistic Anchoring and Mapping

ISH-based spatial transcriptomics and dissociative single-cell RNA sequencing (scRNA-seq) offer complementary strengths: ISH provides spatial coordinates but profiles a limited gene panel, while scRNA-seq captures the full transcriptome but loses spatial context. A central computational challenge is to integrate these modalities by using ISH data as a spatial anchor for scRNA-seq profiles. Tangram, a deep-learning framework published in Nature Methods in 2021 [46], learns a mapping between scRNA-seq cells and spatial locations by optimizing a cosine similarity loss function on shared marker genes, effectively projecting the full scRNA-seq transcriptome onto the spatial coordinate system. Recent refinement strategies have improved Tangram’s consistency and reliability [47], including entropy regularization to prevent diffuse cell mapping and the use of informatically selected gene panels (for example, Spapros) for training [6].

Seurat v5 implements spatial integration workflows [48], including anchor-based label transfer for mapping single-cell references onto spatial measurements. For deconvolution, Seurat can interface with external methods such as RCTD [49] rather than originating them. CytoSPACE extends these approaches [50] by formulating single-cell-to-spatial alignment as a global optimization problem. Additional integration tools—including cell2location for Bayesian cell-type mapping [51], DestVI for variational inference-based deconvolution [52], and PASTE for cross-section alignment [53]—provide complementary analytical perspectives.

#### 4.2.2. The Problem of Sparsity

Even with improved chemistry and imaging, high-plex ISH datasets remain sparse relative to the underlying transcriptome. Many genes are detected in only a fraction of the cells in which they are expressed, and low-abundance transcripts are particularly vulnerable to technical dropout. This sparsity reflects a combination of probe accessibility, hybridization efficiency, amplification efficiency, imaging thresholds and decoding uncertainty. Computational methods can partially mitigate these effects by borrowing information across neighboring spatial units or related cell states, as in BayesSpace for spatial-domain refinement [54], but imputation and smoothing can also blur true boundaries if not interpreted carefully. For this reason, downstream biological conclusions benefit from retaining detection-confidence information and distinguishing absence of evidence from evidence of absence.

#### 4.2.3. Spatial Domains and Spatially Variable Genes

A complete computational workflow also requires methods that identify spatial domains and spatially variable genes (SVGs). Domain-detection tools such as SpaGCN, BANKSY and STAGATE use spatial adjacency, histology or graph neural networks to identify tissue compartments that may not be apparent from expression alone [55,56,57]. SVG methods ask a related but distinct question: which genes show non-random spatial patterning? SPARK, nnSVG and related methods differ in their null hypotheses, statistical models and treatment of cell-type composition [58,59]. Yan et al. [60] categorized 34 SVG methods into overall, cell-type-specific and spatial-domain-marker approaches, underscoring that SVG calls are not interchangeable across methods. For ISH-based data, targeted panels, dropout, uneven detection efficiency and decoding uncertainty can all influence SVG inference; biological interpretation therefore needs to distinguish a genuinely spatial regulatory program from a cell-type marker that appears spatial only because the corresponding cell type is spatially localized.

### 4.3. Cell-Cell Communication Inference

The spatial resolution afforded by ISH-based methods has pushed computational tools to incorporate cellular coordinates when inferring intercellular communication. Unlike cell-cell communication (CCC) methods applied to dissociated scRNA-seq data, which infer potential interactions without direct spatial context, spatial CCC tools can incorporate physical distance, neighborhood definitions and tissue barriers. CellChat provides a widely used ligand-receptor framework [61], COMMOT uses collective optimal transport to account for competition among ligand-receptor species while respecting spatial constraints [62], and NicheNet links upstream ligands to downstream target-gene programs [63]. Additional tools such as stLearn, NICHES and SpaTalk illustrate the diversity of modeling choices, including neighborhood enrichment, graph construction and receptor-ligand-target inference [64,65,66]. Because CCC analysis remains hypothesis-generating, inferred interactions are most convincing when supported by spatial colocalization, receptor availability, perturbation, protein-level validation or functional readouts.

### 4.4. Standardization and Quality Metrics

A milestone in the maturation of the field is the Spatial Touchstone (ST) initiative, published in Nature Biotechnology in December 2025 [44]. This multi-institute effort generated a standardized reference dataset comprising six tissue types processed at multiple global sites on both Xenium and CosMx platforms. The initiative assessed reproducibility, sensitivity, dynamic range, signal-to-noise ratio, false discovery rate, and concordance with single-cell profiling, and produced two key community resources: Standardized Operating Procedures (STSOPs) and SpatialQM, an open-source software package for automated quality metric evaluation. The accompanying Spatial Touchstone Portal provides a web-based repository of over 254 spatial profiles against which researchers can benchmark their own datasets.

Complementary to platform-specific standardization, the Sopa pipeline [67] provides a platform-agnostic workflow for processing spatial omics data, supporting Xenium, Visium HD, MERSCOPE, CosMx, and other platforms through a unified SpatialData structure. Similarly, the SpatialData framework [68], built on the scverse ecosystem, provides standardized data containers for multi-modal spatial datasets, enabling interoperability between analytical tools and facilitating the development of community benchmarks. Efforts to define community-wide data sharing standards further support this interoperability [69].

## 5. Biological Frontiers

### 5.1. Neuroscience and Brain Mapping

The mammalian brain, with its extraordinary cellular diversity and intricate spatial organization, has been a primary area of application for high-plex ISH technologies. MERFISH- and seqFISH-derived atlases have shown how transcriptomic neuronal and glial subtypes are organized within cortical layers, hippocampal subfields, hypothalamic nuclei and molecularly defined neighborhoods, providing the anatomical context that dissociative single-cell sequencing necessarily discards. With this context, taxonomic clusters identified by scRNA-seq can be asked whether they correspond to spatially coherent circuit components, developmental trajectories or dispersed functional states. Barcode-based in situ sequencing approaches such as BARseq sit at the boundary of conventional ISH but illustrate how spatial readout can link projection or barcode identity to gene expression and anatomical position, which is particularly powerful for relating neuronal identity to circuit organization [70,71]. ISH-based methods also recover subcellular information about RNA localization and compartmentalization that dissociative sequencing cannot capture, and digital HCR imaging has been used to resolve sparse neuropeptide or neuromodulator transcripts in thick autofluorescent brain tissue, where targeted high-sensitivity readout is more reliable than transcriptome-scale sequencing [72].

### 5.2. Cancer and Immuno-Oncology

In cancer and immuno-oncology, imaging-based ISH adds value by preserving the topology of the tumor microenvironment (TME): where malignant states sit relative to immune exclusion zones, fibroblast barriers, vascular niches and tertiary lymphoid structures. Bulk RNA-seq can identify inflamed or immune-suppressed tumors, and scRNA-seq can catalog cell states, but neither directly shows whether ligand-expressing tumor or stromal cells are physically positioned to affect receptor-expressing immune cells. ISH-based platforms are particularly useful for FFPE tumor specimens from clinical archives and for same-section RNA-protein workflows, such as Xenium RNA followed by Xenium Protein subpanels (10x Genomics, 2026) or CosMx WTx RNA followed by multiplex protein detection—which enables integrated spatial phenotyping without relying on cross-section registration [7]. Spatially informed Cell–Cell Communication analyses, including COMMOT [62] and CellChat-based workflows [61,73], have been applied to map ligand–receptor interactions within the TME and to highlight communication circuits that can then be tested by protein staining, genetic perturbation or correlation with clinical outcome.

### 5.3. Developmental Biology and Non-Model Organisms

In developmental biology and in species that lack mature single-cell atlases, ISH remains the most direct way to link gene expression to morphology, lineage position and developmental timing. HCR in particular has been widely adopted across embryos, larvae, plants, cnidarians and other fluorescence-rich specimens, in part because its enzyme-free amplification and split-initiator background suppression are well suited to whole-mount preparations where enzymatic protocols are difficult to control [14,74,75]. For non-model organisms, spatial ISH effectively bridges comparative genomics and organismal biology, by showing whether an orthologous gene is deployed in the same tissue, a shifted cell layer or a lineage-specific structure. Open-source probe design pipelines such as OligoMiner [8] have further lowered the barrier for applying HCR-FISH and related approaches in species.

### 5.4. Functional Genomics: Spatial CRISPR Screens

Image-based ISH is also reshaping functional genomics by attaching tissue coordinates to pooled perturbation experiments. Conventional single-cell CRISPR screens link perturbations to transcriptomic phenotypes but lose information about local tissue architecture, clone organization and microenvironmental response. Perturb-RAEFISH directly images guide RNA (gRNA) sequences in situ alongside spatial transcriptomic output [23], and the earlier spatial CRISPR genomics work of Dhainaut et al. [33] identified regulators of the tumor microenvironment in tissue context; Perturb-FISH and related targeted imaging strategies offer further designs in which perturbation identity, selected transcripts and tissue position are jointly measured. Together, these approaches make it possible to ask whether the effect of a perturbation is cell-autonomous or contingent on local position—for example, on proximity to an invasive front, an immune interface, a developmental boundary or a signaling niche. The biological resolution of these screens still depends on accurate guide detection, panel design and cell segmentation, particularly in dense tissues where misassigned guides or boundaries can mimic real spatial effects.

## 6. Discussion: Challenges, Opportunities, and the Road Ahead

Modern ISH has developed into a broad methodological spectrum rather than a single class of assays. At one end, HCR, RNAscope, SABER-FISH and related targeted approaches remain essential for robust visualization of selected transcripts in intact cells and tissues. At the other end, MERFISH, seqFISH, RAEFISH and commercial imaging platforms extend the same basic logic of sequence-specific recognition into high-plex spatial transcriptomics. The practical value of these methods is therefore not determined by gene-panel size alone. It depends on how each workflow balances probe specificity, signal amplification, tissue compatibility, imaging burden, optical crowding, segmentation accuracy and decoding confidence. For a small number of candidate transcripts in FFPE tissue or whole-mount specimens, a highly validated low-plex method may be more appropriate than a transcriptome-scale platform. Conversely, atlas construction, spatial perturbation screens and cell-neighborhood analysis require higher-plex approaches with explicit error control and reproducible computational processing.

A central challenge for the field is reproducibility across platforms, laboratories and tissue types. Imaging-based spatial transcriptomics involves many coupled steps—fixation, permeabilization, probe hybridization, amplification, cyclic imaging, signal removal, image registration, segmentation and molecule-to-cell assignment. Differences at any step can affect transcript counts, inferred cell states and downstream spatial analyses. Recent benchmarking and standardization efforts, including Spatial Touchstone and SpatialQM, are important because they shift the field from platform-specific performance claims toward shared metrics for sensitivity, specificity, false discovery, reproducibility and cross-run comparability. However, benchmark results should be interpreted within their experimental context. Platform rankings can change with tissue type, panel design, fixation history, segmentation strategy, software version and the choice of orthogonal reference. Thus, future comparisons will be most useful when they report not only aggregate performance, but also probe sequences, hybridization conditions, imaging parameters, decoding thresholds, segmentation settings and quality-control outputs.

The evidence base also remains uneven across the technologies reviewed here. Some methods, such as HCR v3.0, RNAscope, MERFISH and seqFISH, have accumulated extensive independent use across biological systems. Others, including RAEFISH, HCR-Cat, HCR-Multi, RT&T-AMP-MERFISH and several emerging commercial workflows, are promising but still supported by a smaller number of studies or by early-stage reports. This does not diminish their importance, but it changes how their performance should be interpreted. For example, RAEFISH demonstrates an important route toward transcriptome-scale image-based profiling through pooled, amplifiable probe libraries and RCA-based decoding, but its practical sensitivity, robustness and cost structure will need broader validation across tissues and laboratories. Similarly, vendor-reported panel size, workflow compatibility or protein co-detection should be distinguished from independently benchmarked performance. A mature field will require both technical innovation and transparent evidence categories.

Cost and accessibility are equally important. Commercial platforms provide integrated instruments, standardized reagents, automated fluidics and supported analysis pipelines, making high-plex spatial profiling more accessible to laboratories without extensive engineering expertise. These advantages come with high capital and reagent costs, closed chemistries and limited flexibility. Open or academic implementations can reduce entry costs and allow protocol modification, but often require substantial expertise in probe design, microfluidics, microscopy, image processing and bioinformatics. New probe-library strategies, including RAEFISH-style pooled synthesis, may lower one component of the cost of transcriptome-scale imaging, but total experiment cost still includes tissue processing, fluidics, imaging time, data storage, computational decoding, labor and failed runs. Open analytical ecosystems such as Sopa, Squidpy, SpatialData and the broader scverse framework can help reduce computational barriers, provided that input data and metadata are sufficiently standardized.

Several technical directions are likely to shape the next phase of ISH-based spatial biology. Same-section multi-omics is becoming increasingly feasible, with workflows that combine RNA imaging with protein detection and, in some cases, chromatin or genomic-locus readout. The main challenge is not conceptual but chemical and statistical: sequential assays must preserve RNA, antigenicity and chromatin structure across many cycles, while analysis methods must integrate modalities with different spatial supports and noise models. Isoform-resolved and non-coding-RNA spatial imaging represent another frontier. Methods such as RT&T-AMP-MERFISH indicate that spatial isoform detection at large scale is possible [76], and RAEFISH shows that long non-coding RNAs can be incorporated into transcriptome-scale probe libraries, but these capabilities remain far less widely validated than gene-level RNA detection. Three-dimensional and expansion-based approaches address a related limitation: tissue architecture and molecular organization are inherently three-dimensional, whereas most current experiments remain two-dimensional. Serial-section alignment and volumetric imaging strategies, surveyed in a recent comprehensive review [77], together with expansion-based methods such as ExSeq [78], can improve access to 3D organization and reduce molecular crowding, but they add new demands on sample processing, molecular retention and spatial registration.

Biological interpretation will increasingly depend on how these technical choices are matched to the question being asked. Spatial ISH is particularly powerful when location changes the meaning of gene expression: when rare transcripts identify anatomical niches, when neighboring cell states define immune or developmental microenvironments, when subcellular RNA localization informs function, or when perturbation effects depend on tissue position. At the same time, spatial proximity and co-expression do not by themselves establish mechanism. Cell-cell communication inference, spatial-domain detection and perturbation mapping should therefore be treated as hypothesis-generating unless supported by orthogonal validation, protein-level evidence, perturbation experiments or functional readouts. In this sense, the future of ISH-based spatial transcriptomics will depend not only on higher plex or larger tissue coverage, but on workflows that make the complete chain from molecular detection to biological interpretation transparent and reproducible.

Together, the methods reviewed here trace the evolution of ISH from targeted molecular visualization to a versatile framework for in situ detection and spatially resolved transcriptomic analysis. The field is now moving from proof-of-principle demonstrations toward routine, quantitative and comparative use. Achieving this transition will require continued progress in chemistry, imaging and computation, but also shared reference materials, reporting standards, confidence metrics and interoperable data structures. With such infrastructure, modern ISH methods can become not only powerful tools for individual studies, but cumulative technologies for mapping molecular organization across tissues, organisms and disease states.

## Figures and Tables

**Table 1:** Molecular logic and application of major ISH signal-amplification strategies.

Method	Strength	Caveats	Use Cases	Situations Requiring Caution	Evidence Base
HCR v3.0	Enzyme-free amplification; split-initiator design suppresses background; works well in whole mounts and many non-model organisms.	Sensitivity may still be insufficient for very short or very rare targets; hairpin quality, tissue penetration and autofluorescence can limit performance.	Candidate-gene validation, embryos, thick or whole-mount samples, non-model organisms.	Transcriptome-scale profiling without extensive imaging rounds; sensitivity may be insufficient for very short or very rare targets; background signal may accumulate if hairpin quality or tissue penetration is suboptimal.	Peer-reviewed method with broad biological use.
HCR-Cat/HCR-Multi	Reported signal enhancement through catalytic or immunological amplification; useful for sparse targets in the original demonstrations.	More steps and reagents; potential diffusion of tyramide signal; antibody/HRP performance and quenching become critical.	Low-abundance targets and highly autofluorescent or thick samples.	Quantitative cross-target comparisons require careful normalization and controls; tyramide or catalytic amplification steps may introduce signal diffusion or variability.	Emerging; currently supported primarily by preprint-stage evidence, with broad cross-laboratory validation still needed.
RNAscope/bDNA	High robustness in formalin-fixed, paraffin-embedded (FFPE) tissue; strong signal amplification; widely used in pathology-oriented workflows.	Commercial/proprietary design limits transparency; panel size is modest; puncta can saturate for highly expressed genes; cost can be high.	Clinical archives, low-to-moderate plex validation, FFPE pathology specimens.	Open-ended transcriptome-scale discovery or low-cost large-panel screening; high-expression puncta may saturate, and careful panel and tissue selection is required.	Peer-reviewed and commercial evidence; product specifications are version- and date-dependent.
SABER-FISH/PER	Programmable concatemers provide tunable brightness and exchange-based multiplexing.	Polymerase-generated concatemers must be quality controlled; long scaffolds can increase steric burden and nonspecific background.	Moderate-plex imaging where signal strength must be tuned.	Highly degraded samples or workflows requiring minimal enzymatic preparation; long concatemer scaffolds can increase steric hindrance and nonspecific background.	Peer-reviewed academic method.
RCA/padlock-based FISH	Digital rolony-like amplicons; ligation provides sequence specificity; compatible with iterative decoding and in situ sequencing.	Reverse transcription and ligation inefficiency can cause false negatives; amplicon size can crowd dense regions; probe synthesis can be costly.	Digital molecule counting, platforms using padlocks, barcode-based spatial profiling.	Very dense transcriptomes without optical-crowding mitigation; reverse transcription and ligation inefficiencies may lead to false negatives; amplicon size may crowd imaging regions.	Peer-reviewed method family; platform implementations vary.

The table summarizes typical use cases and caveats; performance varies with probe design, sample preparation, imaging conditions and analysis pipeline. FFPE, formalin-fixed, paraffin-embedded; HCR, hybridization chain reaction; HRP, horseradish peroxidase; PER, primer exchange reaction; RCA, rolling circle amplification.

**Table 2:** Benchmark-aware comparison of selected commercial platforms and RAEFISH.

Platform/Method	Evidence and Benchmark Context	Target Panel/Scale	Reported Quantitative Signals	Practical Interpretation/Considerations
Xenium	Commercial padlock/RCA imaging platform; evaluated in peer-reviewed FFPE benchmarks and updated through vendor releases.	289–339 genes in Ozirmak Lermi et al. [36]; Xenium 5K/Prime panels in later benchmarks and product versions.	Ozirmak Lermi et al. [36]: FDR < 0.09% using negative/blank controls; bulk RNA-seq correlation R, 0.62–0.66; DSP WTA correlation R, 0.68–0.80. Wang et al.: higher matched-gene transcript counts without apparent specificity loss.	Strong FFPE performance in several benchmarks, but rankings depend on tissue type, panel version, segmentation mode and software version.
CosMx	Commercial smFISH/barcoding-derived imaging platform; benchmarked against Xenium, MERFISH and orthogonal RNA/protein data.	1000-plex in Ozirmak Lermi et al. [36]; 6K and WTx offerings reported in product literature and recent studies.	Ozirmak Lermi et al. [36]: FDR 10–11% in ICON TMAs and 5.8–6.2% in MESO TMAs; DSP WTA correlation R, 0.84–0.85; CosMx-Xenium correlation R, 0.86–0.88 on shared genes.	Large panels and RNA-protein workflows are attractive for tumor atlasing, but field-of-view (FOV) selection, segmentation, tissue age and background correction can strongly shape results.
MERSCOPE/MERFISH	Commercial implementation of MERFISH principles plus academic MERFISH lineage; benchmarked in FFPE tumor TMAs.	500 genes in the Ozirmak Lermi [36] immuno-oncology comparison; commonly hundreds to approximately 1000 genes depending on assay design.	Ozirmak Lermi et al. [36]: FDR 4.4–6% in ICON and 4.8% in MESO1 using blank probes; cells retained after filtering ranged from 22.6% to 64.4%; DSP WTA correlation R, 0.67–0.77.	Error-robust barcoding is conceptually strong, but practical performance depends heavily on tissue quality, chemistry generation, panel design and filtering thresholds.
G4X	Commercial cyclic sequencing/imaging platform; some capabilities are still primarily supported by vendor or early-access information.	Hundreds of RNA targets plus reported protein add-on; immune-receptor readout is a differentiating claim.	Independent, peer-reviewed head-to-head quantitative benchmarking remains limited compared with Xenium, CosMx and MERSCOPE.	An emerging platform whose performance will require broader independent benchmarking as applications expand.
RAEFISH	Academic reverse-padlock/RCA method reported in peer-reviewed literature; not a commercial instrument.	Approximately 23,000 probed genes in human; >20,000-gene whole-transcriptome-scale probe sets.	Original study: 3749 RNA molecules and 1287 distinct genes per A549 cell on average; bulk RNA-seq concordance r, 0.66; inter-replicate r, 0.85; reported probe-library cost approximately $158, excluding imaging and computation.	A major design advance for transcriptome-scale image-based profiling; current data indicate partial per-cell recovery and motivate broader independent replication.
Sequencing-based comparators	Included only where benchmarks compare spatial capture modalities with imaging-based platforms.	Visium v1/v2/CytAssist, Visium HD and Stereo-seq appear in Cervilla et al. and Ren et al. comparisons.	Cervilla et al. evaluated transcript and unique molecular identifier (UMI) detection [38], gene-histology concordance, cell-type recovery and protein-panel integration across six FFPE cancer types.	These platforms are outside the main ISH scope but help define trade-offs between transcriptome breadth, spatial resolution, tissue area and cost.

FFPE, formalin-fixed, paraffin-embedded; FDR, false discovery rate; DSP WTA, GeoMx Digital Spatial Profiler whole-transcriptome atlas assay; RCA, rolling circle amplification; WTx, whole transcriptome. Numerical values summarize the cited benchmark contexts and are not intended to generalize across all tissues, panels, software versions or sample-preparation workflows. RAEFISH is an academic method rather than a commercial instrument.

## Data Availability

No new data were created or analyzed in this study. Data sharing is not applicable to this article.

## Glossary

Abbreviations used in this review: bDNA, branched DNA; CCC, cell-cell communication; CRISPR, clustered regularly interspaced short palindromic repeats; DSP WTA, Digital Spatial Profiler whole-transcriptome atlas; FDR, false discovery rate; FF, fresh frozen; FFPE, formalin-fixed paraffin-embedded; FOV, field of view; gRNA, guide RNA; HCR, hybridization chain reaction; HRP, horseradish peroxidase; ISH, in situ hybridization; LNA, locked nucleic acid; PER, primer exchange reaction; PNA, peptide nucleic acid; QC, quality control; RCA, rolling circle amplification; SAM, Segment Anything Model; SBL, sequencing by ligation; scRNA-seq, single-cell RNA sequencing; smFISH, single-molecule fluorescence in situ hybridization; SVG, spatially variable gene; TMA, tissue microarray; TME, tumor microenvironment; TSA, tyramide signal amplification; UMI, unique molecular identifier; WTx, whole transcriptome.
